# Supplementary figures and images for: Hippo signaling pathway activation during SARS-CoV-2 infection contributes to host antiviral response
Source: PLoS Biol. 2022 Nov 8;20(11):e3001851. doi: 10.1371/journal.pbio.3001851 (PMC9642871; doi:10.1371/journal.pbio.3001851)

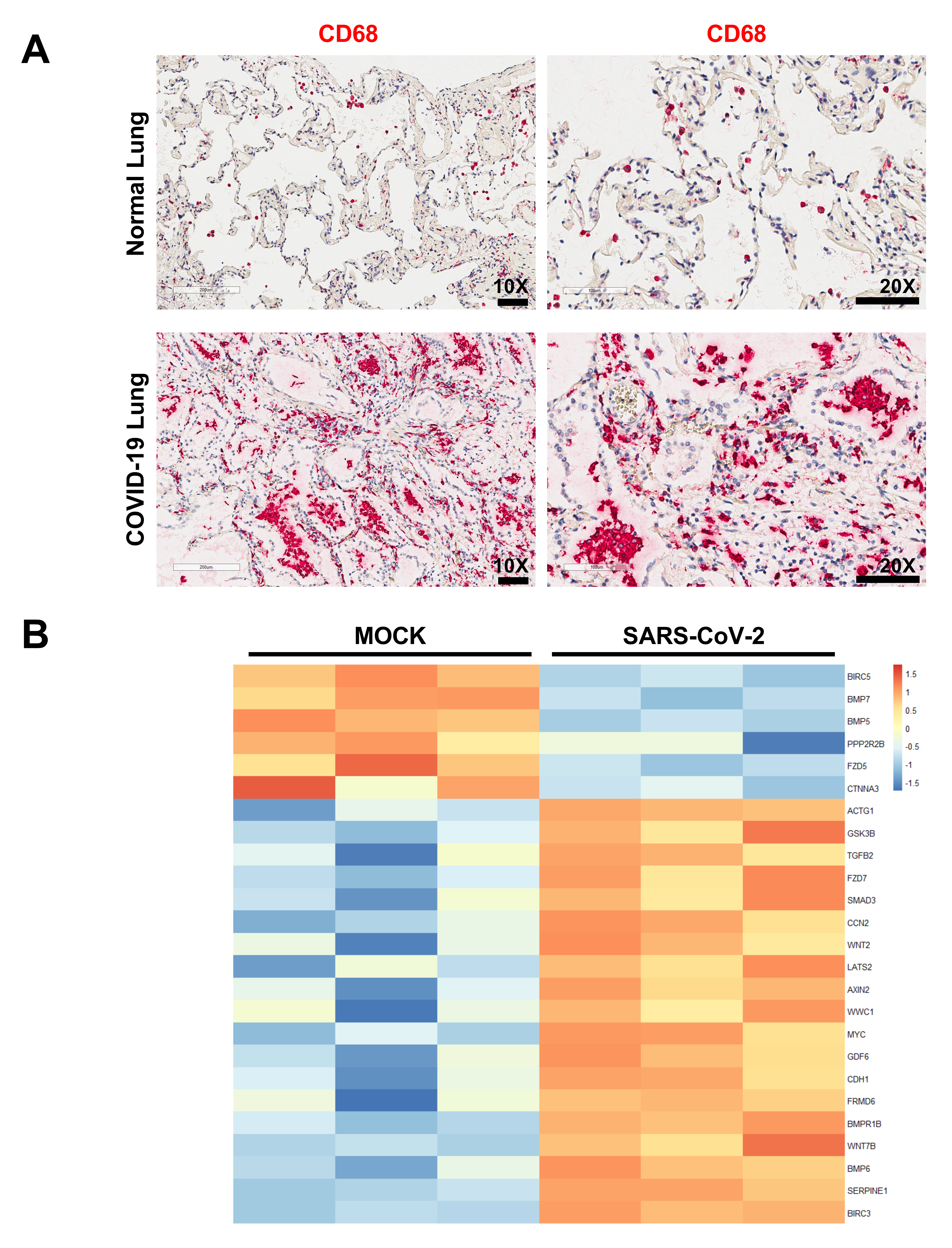

Supplement: S1 Fig — (A) Immunohistochemistry of COVID-19 lung autopsy tissue shows heavy infiltration of CD68-positive inflammatory cells (red) (scale bar: 100 μm). (B) Heatmap depicting Z scores as expression levels of the 25 differentially expressed genes (p < 0.01) involved in Hippo signaling pathway of SARS-CoV-2-infected hPSC-cardiomyocytes relative to uninfected cells at 3 dpi. Blue and red colors represent 6 down-regulated and 19 up-regulated genes, respectively. These differentially expressed genes in the Hippo signaling pathway were identified using the KEGG pathway database. The gene expression data was retrieved at Gene Expression Omnibus with accession number GSE150392. Individual quantitative observations that underlie the data summarized can be located under the Supporting Information File as S4 Data. (TIF) [file pbio.3001851.s001.tif]

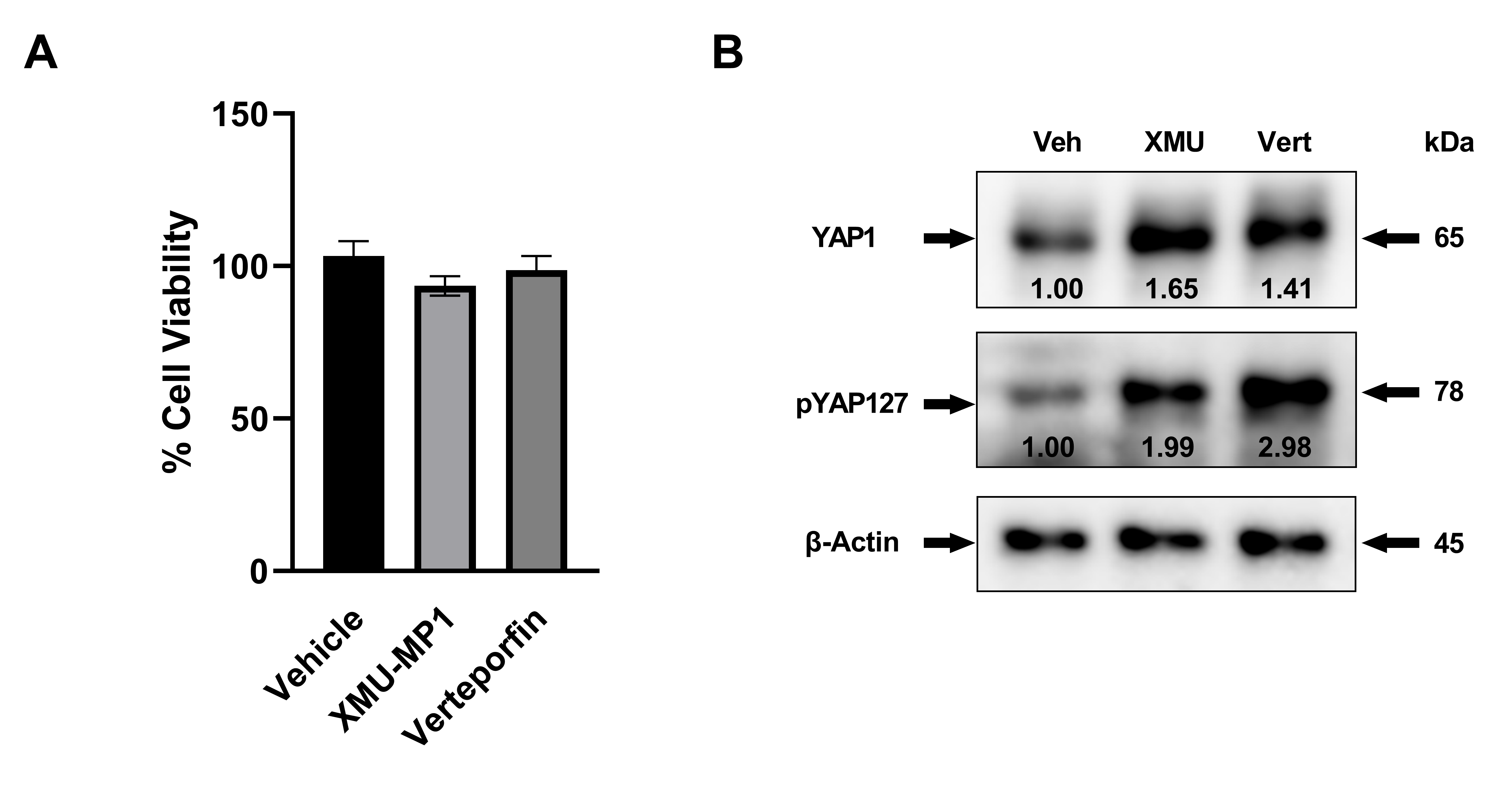

Supplement: S2 Fig — (A) Graph shows the percent cytotoxicity of Calu-3 cells 72 hours post-treatment with DMSO (Vehicle), XMU-MP1 (10 μm), and Verteporfin (1 μm). CellTiter-Glo Luminescent Cell Viability Assay was performed as per the manufacturer (Promega, USA) recommendation. Individual quantitative observations that underlie the data summarized can be located under the Supporting Information File as S5 Data. (B) Western blot analysis shows total and phosphorylated YAP at 72 hours post treatment with indirect and direct acting inhibitors, XMU-MP-1 and Verteporfin, respectively. Note: XMU-MP-1 treatment enhances YAP/TAZ level compared to vehicle, whereas Verteporfin increases phosphorylated YAP (S127) levels. Representative data from 2 independent experiments is shown. (TIF) [file pbio.3001851.s002.tif]
