# Supplementary material for: Hippo signaling pathway activation during SARS-CoV-2 infection contributes to host antiviral response
Source: PLoS Biol. 2022 Nov 8;20(11):e3001851. doi: 10.1371/journal.pbio.3001851 (PMC9642871; doi:10.1371/journal.pbio.3001851)
Supplement: S1 Table — (DOCX) [file pbio.3001851.s003.docx]

**SUPPLEMENTAL TABLES**

**Supplementary Table 1:** Reagents or resources used in this study.

| **REAGENT/RESOURCE** | **SOURCE** | **IDENTIFIER** |
| --- | --- | --- |
| **Antibodies** | | |
| Monoclonal anti-SARS-CoV S protein (Similar to 240C) antibody | BEI Resources Repository | Cat#NR-616 |
| Polyclonal anti-SARS coronavirus (antiserum) | BEI Resources Repository | Cat#NR-10361 |
| SARS-CoV-2 (COVID-19) Nucleocapsid antibody [HL344] | GeneTex | Cat#GTX635679 |
| IRF-3 (D6I4C) XP | Cell Signaling Technology | Cat#11904 |
| TBK1/NAK (D1B4) | Cell Signaling Technology | Cat#3504S |
| Phospho-TBK1/NAK (Ser172) | Cell Signaling Technology | Cat#5483S |
| YAP/TAZ (D24E4) | Cell Signaling Technology | Cat# 8418S |
| Phospho-YAP (Ser127) | Cell Signaling Technology | Cat#13008S |
| COVID V-nCoV2019-S probe | ACD | Cat#: 848568 |
| AC TUBULIN | Cell Signaling Technology | Cat#mAb#5335 |
| MUC5AC Mouse | Invitrogen | Cat#MA512178 |
| Anti-Cardiac Troponin T antibody | Abcam | Cat#ab45932 |
| Recombinant Anti-IRF3 (phospho S386) antibody [EPR2346] | Abcam | Cat#ab76493 |
| Cleaved caspase-3 rabbit monoclonal antibody, clone D175 | Cell Signaling Technology | Cat#9661S |
| Goat anti-Mouse IgG (H+L) Cross-Adsorbed Secondary Antibody, Alexa Fluor 555 | Thermo Fisher Scientific | Cat#A21422 |
| IgG (H+L) Cross-Adsorbed Goat anti-Rabbit, Alexa Fluor 488, Invitrogen | Thermo Fisher Scientific | Cat#A11008 |
| IgG (H+L) Cross-Adsorbed Goat anti-Human, Alexa Fluor 488, Invitrogen | Thermo Fisher Scientific | Cat#A11013 |
| Goat anti-Guinea Pig IgG (H+L) Highly Cross-Adsorbed Secondary Antibody, Alexa Fluor 488 | Thermo Fisher Scientific | Cat#A11073 |
| Monoclonal Anti-Beta-Actin, Clone AC-74 produced in mouse | MilliporeSigma | Cat#A2228 |
| Phospho-Stat1 (Tyr701) (58D6) Rabbit mAb | Cell Signaling Technology | Cat#9167S |
| Stat1 (D1K9Y) Rabbit mAb | Cell Signaling Technology | Cat#14994 |
| CD68 | Dako | Cat#m0876 |
| **Bacterial and Virus Strains** | | |
| SARS-Related Coronavirus 2 (SARS-CoV-2), Isolate USA-WA1/2020 | BEI Resources Repository | Cat#NR-52281 |
| SARS-Related Coronavirus 2, Isolate hCoV-19/USA/MD-HP05647/2021 (Lineage B.1.617.2; Delta variant), | BEI Resources Repository | Cat#NR-55672 |
| SHCLNG MISSION shRNA YAP1 Bacterial Clone | Sigma-Aldrich | Cat# TRCN0000107267 |
| SHCLNG MISSION shRNA LATS1 Bacterial Clone | Sigma-Aldrich | Cat# TRCN0000001779 |
| **Chemicals, Peptides, and Recombinant Proteins** | | |
| Regular Fetal Bovine Serum | Corning | Cat#35010CV |
| Eagle's Minimum Essential Medium (MEM) | Corning | Cat#10009CV |
| Penicillin-Streptomycin (10,000 U/mL) | Gibco | Cat#15140122 |
| L-Glutamine (200 mM) | Gibco | Cat#25030081 |
| Puromycin Dihydrochloride | Gibco | Cat#A1113803 |
| PneumaCult-ALI Medium | STEMCELL Technologies | Cat#05021 |
| AR9 BUFFER, 10X | Akoya Biosciences | Cat#AR9001KT |
| Dimethyl sulfoxide | MilliporeSigma | Cat#D2650 |
| RPMI 1640 | Thermo Fisher Scientific | Cat#11875093 |
| B27 supplement with insulin | Thermo Fisher Scientific | Cat#17504044 |
| Methanol (Histological) | Thermo Fisher Scientific | Cat#A433P4 |
| 16% Paraformaldehyde (formaldehyde) aqueous solution | Electron Microscopy Sciences | Cat#15710 |
| Dulbecco's Phosphate-Buffered Salt Solution 1X | Corning | Cat#21030CV |
| Perm/Wash Buffer | BD Biosciences | Cat#554723 |
| DAPI (4',6-Diamidino-2-Phenylindole, Dihydrochloride) | Thermo Fisher Scientific | Cat#D1306 |
| SuperBlock (PBS) Blocking Buffer | Thermo Fisher Scientific | Cat#37515 |
| Bovine Serum Albumin | MilliporeSigma | Cat#A9418 |
| Normal Donkey Serum | Jackson ImmunoResearch | Cat#017-000-121 |
| Normal Goat Serum | Cell Signaling Technology | Cat#5425S |
| Triton-X 100 | MilliporeSigma | Cat#T9284 |
| XMU-MP-1 | MilliporeSigma | Cat# SML2233-5MG |
| Verteporfin | MilliporeSigma | Cat#SML0534-5MG |
| **Commercial Assays** | | |
| Bond Polymer Refine Detection Kit | Leica Microsystems | Cat#DS9800 |
| CellTiter 96 Non-Radioactive Cell Proliferation Assay (MTT) | Promega | Cat#G4000 |
| **Experimental Models: Cell Lines** | | |
| VERO C1008 [Vero 76, clone E6, Vero E6] | ATCC | Cat#CRL-158 |
| Calu-3 | ATCC | Cat#HTB-55 |
| hPSC derived cardiomyocyte | University of California, Los Angeles (Li et al., 2021) | N/A |
| Normal human bronchial epithelial cells | Lonza | N/A |
| **Deposited Data** | | |
| RNA-Seq of Human iPSC-cardiomyocytes infected with SARS-CoV-2 | Gene Expression Omnibus | Accession Number: GSE150392 |
| **Software and Algorithms** | | |
| GraphPad Prism 8 | GraphPad | N/A |
| Multi-Point Tool (Cell Counter) | ImageJ | N/A |

**Reference**

1. Li S, Ma F, Yokota T, et al. Metabolic reprogramming and epigenetic changes of vital organs in SARS-CoV-2-induced systemic toxicity. JCI Insight. 2021;6(2):e145027. Published 2021 Jan 25. doi:10.1172/jci.insight.145027
