# Supplementary material for: Hippo signaling pathway activation during SARS-CoV-2 infection contributes to host antiviral response
Source: PLoS Biol. 2022 Nov 8;20(11):e3001851. doi: 10.1371/journal.pbio.3001851 (PMC9642871; doi:10.1371/journal.pbio.3001851)

Figure 1 Raw blots

(Equipment: ChemiDoc MP, Software: Image Lab Version 6.1.0 build 7)
Protein Marker: Page Ruler Prestained Protein Ladder, 10 to 180 kDa.

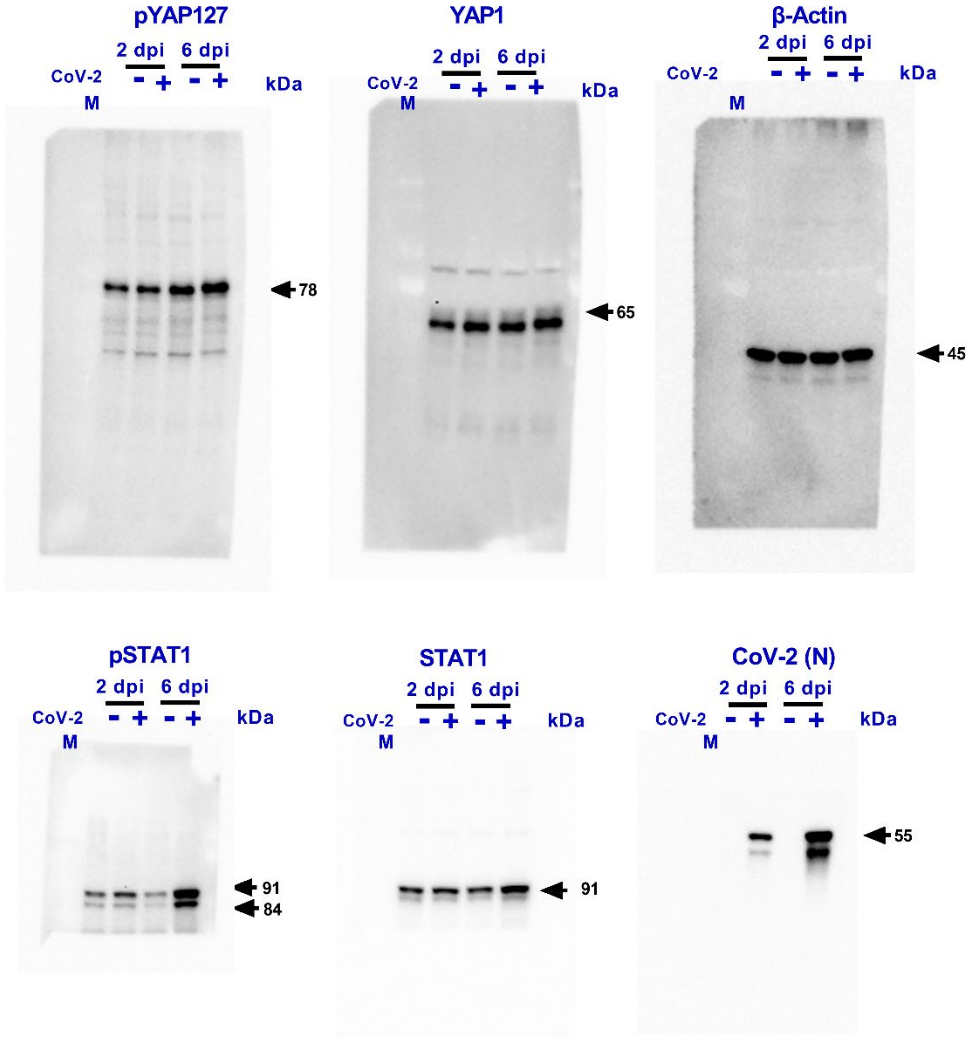

Figure 2 Raw blots

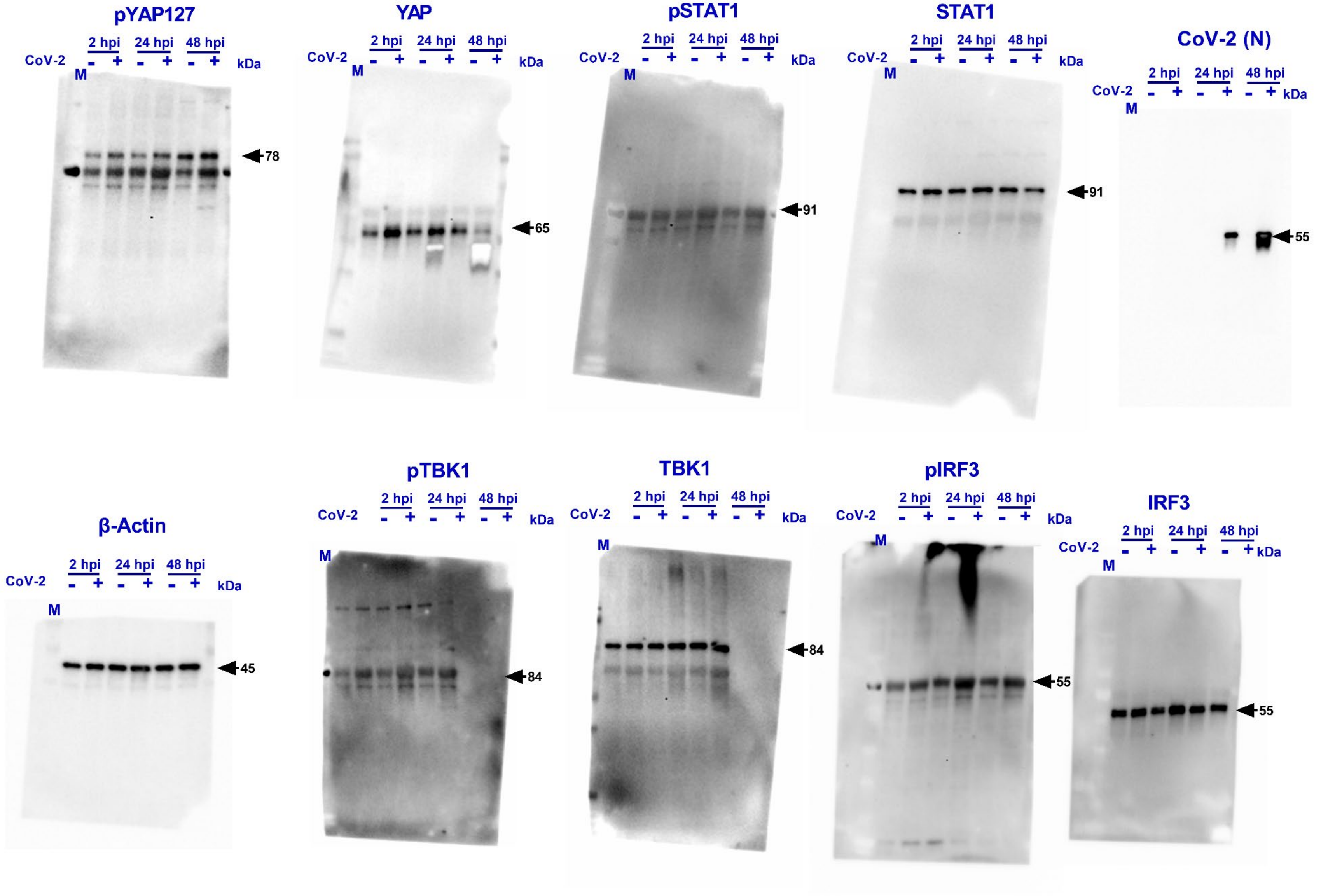

Figure 3B Raw blots

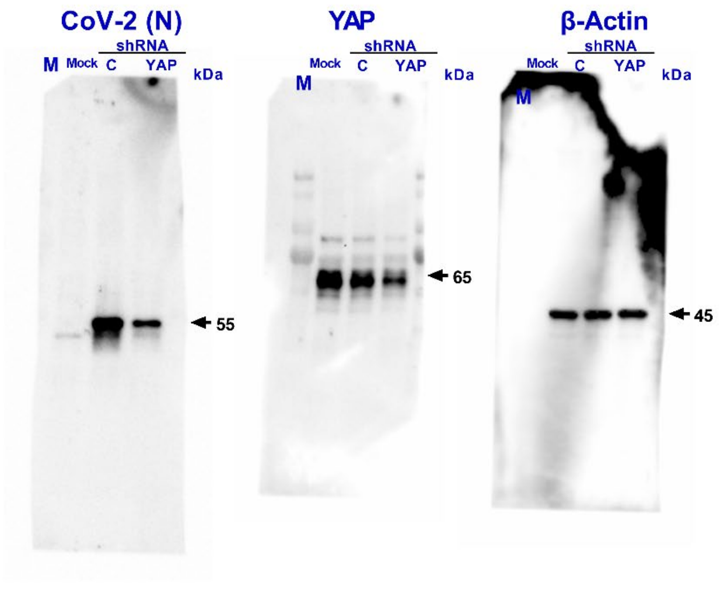

Figure 3F Raw blots

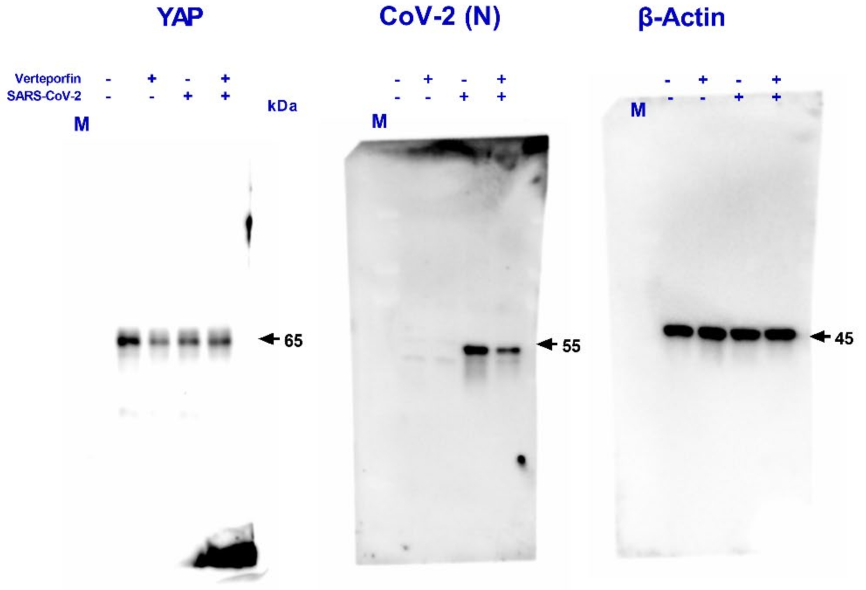

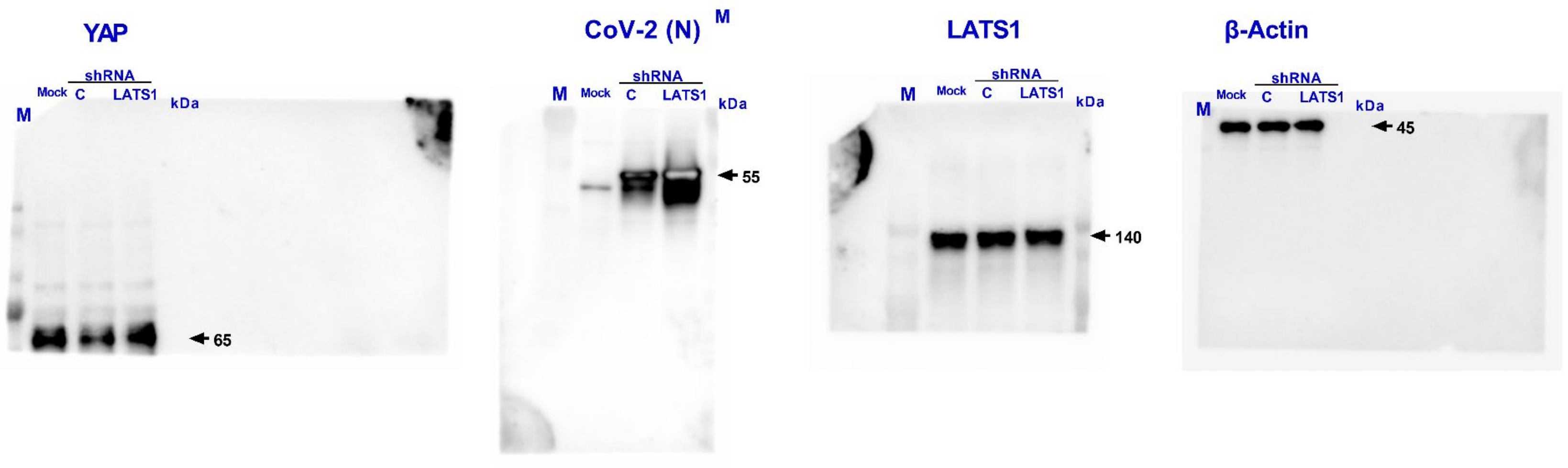

Supplementary Figure 2 Raw blots

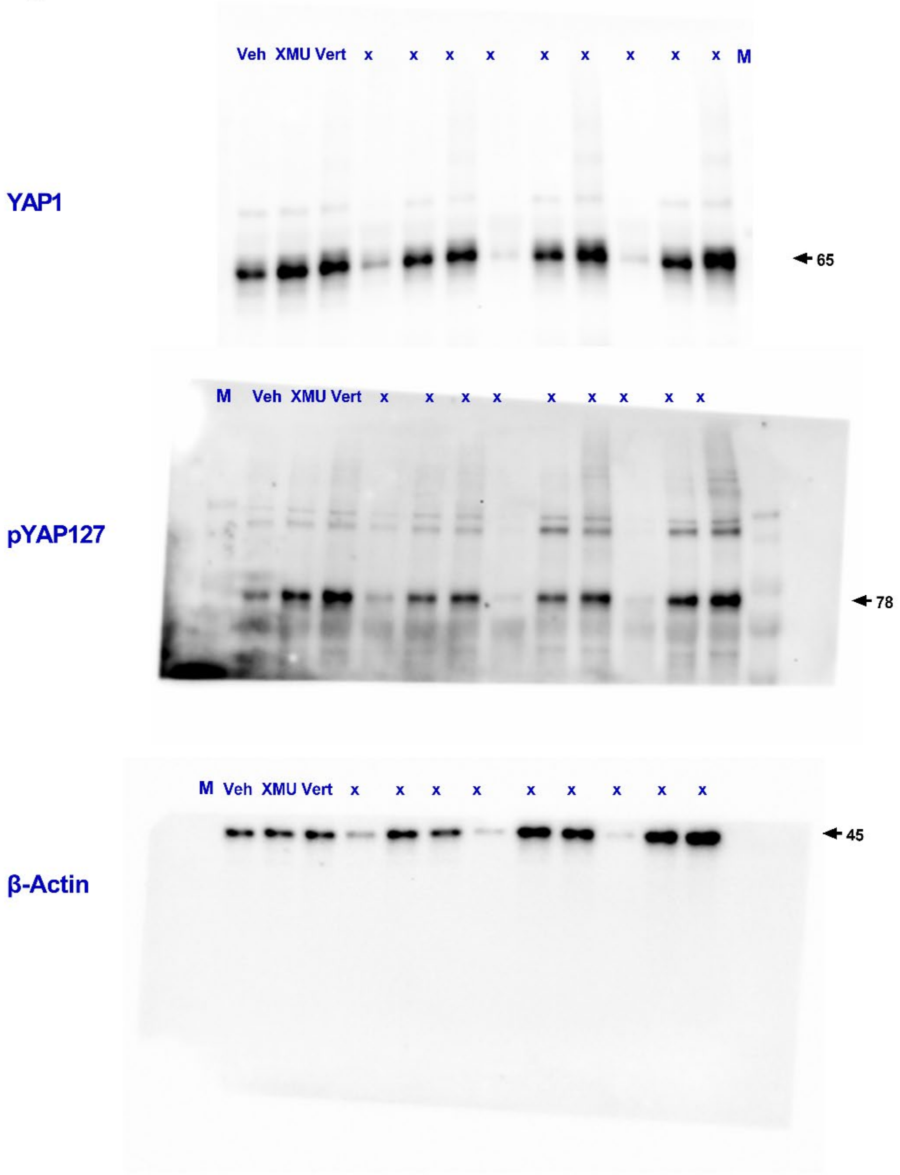

Supplement: S1 Raw Material — (PDF) [file pbio.3001851.s004.pdf]
